# Artificial intelligence in vaccine research and development: an umbrella review

**DOI:** 10.3389/fimmu.2025.1567116

**Published:** 2025-05-08

**Authors:** Rabie Adel El Arab, May Alkhunaizi, Yousef N. Alhashem, Alissar Al Khatib, Munirah Bubsheet, Salwa Hassanein

**Affiliations:** ^1^ Almoosa College of Health Sciences, Alhasa, Saudi Arabia; ^2^ Pediatric Department, Almoosa Specialist Hospital, Alhasa, Saudi Arabia; ^3^ Department of Community Health Nursing, Cairo University, Cairo, Egypt

**Keywords:** artificial intelligence, machine learning, vaccine development, epitope prediction, regulatory frameworks, public acceptance, pandemic preparedness

## Abstract

**Background:**

The rapid development of COVID-19 vaccines highlighted the transformative potential of artificial intelligence (AI) in modern vaccinology, accelerating timelines from years to months. Nevertheless, the specific roles and effectiveness of AI in accelerating and enhancing vaccine research, development, distribution, and acceptance remain dispersed across various reviews, underscoring the need for a unified synthesis.

**Methods:**

We conducted an umbrella review to consolidate evidence on AI’s contributions to vaccine discovery, optimization, clinical testing, supply-chain logistics, and public acceptance. Five databases were systematically searched up to January 2025 for systematic, scoping, narrative, and rapid reviews, as well as meta-analyses explicitly focusing on AI in vaccine contexts. Quality assessments were performed using the ROBIS and AMSTAR 2 tools to evaluate risk of bias and methodological rigor.

**Results:**

Among the 27 reviews, traditional machine learning approaches—random forests, support vector machines, gradient boosting, and logistic regression—dominated tasks from antigen discovery and epitope prediction to supply‑chain optimization. Deep learning architectures, including convolutional and recurrent neural networks, generative adversarial networks, and variational autoencoders, proved instrumental in multiepitope vaccine design and adaptive clinical trial simulations. AI‑driven multi‑omic integration accelerated epitope mapping, shrinking discovery timelines by months, while predictive analytics optimized manufacturing workflows and supply‑chain operations (including temperature‑controlled, “cold‑chain” logistics). Sentiment analysis and conversational AI tools demonstrated promising capabilities for real‑time monitoring of public attitudes and tailored communication to address vaccine hesitancy. Nonetheless, persistent challenges emerged—data heterogeneity, algorithmic bias, limited regulatory frameworks, and ethical concerns over transparency and equity.

**Discussion and implications:**

These findings illustrate AI’s transformative potential across the vaccine lifecycle but underscore that translating promise into practice demands five targeted action areas: robust data governance and multi‑omics consortia to harmonize and share high‑quality datasets; comprehensive regulatory and ethical frameworks featuring transparent model explainability, standardized performance metrics, and interdisciplinary ethics committees for ongoing oversight; the adoption of adaptive trial designs and manufacturing simulations that enable real‑time safety monitoring and in silico process modeling; AI‑enhanced public engagement strategies—such as routinely audited chatbots, real‑time sentiment dashboards, and culturally tailored messaging—to mitigate vaccine hesitancy; and a concerted focus on global equity and pandemic preparedness through capacity building, digital infrastructure expansion, routine bias audits, and sustained funding in low‑resource settings.

**Conclusion:**

This umbrella review confirms AI’s pivotal role in accelerating vaccine development, enhancing efficacy and safety, and bolstering public acceptance. Realizing these benefits requires not only investments in infrastructure and stakeholder engagement but also transparent model documentation, interdisciplinary ethics oversight, and routine algorithmic bias audits. Moreover, bridging the gap from in silico promise to real‑world impact demands large‑scale validation studies and methods that can accommodate heterogeneous evidence, ensuring AI‑driven innovations deliver equitable global health outcomes and reinforce pandemic preparedness.

## 1 Introduction

Recent global health crises, particularly the COVID-19 pandemic, have underscored the urgency of fast-tracking vaccine development, optimization, and distribution (1).Traditional vaccine research and development (R&D) pipelines, spanning several years or even decades, are challenged by complex processes such as antigen discovery, epitope prediction, adjuvant formulation, and rigorous clinical trial designs (2–4). While the speed and scale of COVID-19 vaccine development have demonstrated the promise of artificial intelligence (AI) in modern immunology (5–7), this acceleration resulted from multiple convergent factors: massive funding, preexisting mRNA platforms, overlapping trial phases, and global collaboration—not solely from AI. Nevertheless, AI-based methods—from rapid epitope mapping to adaptive clinical trial designs—have helped shorten certain phases from years to months, illustrating a potential paradigm shift in how vaccines are conceived, tested, and produced (8–12).

Despite these advances, the integration of AI within the vaccine R&D continuum faces ongoing barriers: heterogeneity of immunological datasets, ethical and regulatory uncertainties, and interpretability issues in advanced AI models (13, 14). As global health initiatives stress equitable access and preparedness for future pandemics (15, 16), researchers and policymakers need a robust, evidence-based appraisal of AI’s contributions—balanced with realistic expectations about its limitations. Regulatory and ethical concerns loom large, including transparency, fairness, and explainability of complex AI algorithms. Regulators such as the FDA and EMA are formulating guidelines for safe and effective AI adoption in healthcare contexts, though standardized frameworks for AI in vaccinology are still emerging (17–19). In parallel, issues of intellectual property, data privacy, and consent frameworks necessitate careful navigation to maintain public trust in AI solutions (20).

Although various systematic and narrative reviews have explored AI’s role in vaccine research, development, and dissemination, the evidence base is scattered across different disease targets, computational approaches, and review types. A high-level synthesis of these disparate findings is needed to unify insights, highlight overarching trends, and pinpoint persistent knowledge gaps. We build on existing large-scale reviews but extend the discussion to cover regulatory, logistical, and equity-focused perspectives that are often underrepresented. By integrating multiple sources, this umbrella review aims to guide strategic decisions on high-value AI tools, ethical best practices, and regulatory alignment in vaccine development.

### 1.1 Aim

The aim of this umbrella review is to synthesize the existing evidence on the role of artificial intelligence (AI) in accelerating and enhancing vaccine research, development, distribution, and acceptance.

### 1.2 Research questions

1. What specific AI techniques are most commonly applied in vaccine research and development?
2. How effective are AI-driven methodologies in enhancing various stages of vaccine development compared to traditional approaches?
3. What are the primary ethical, logistical and regulatory challenges associated with integrating AI in vaccine research and distribution?

## 2 Methods

### 2.1 Study design

This umbrella review was conducted to consolidate high-level evidence on AI in vaccine research, development, distribution, and acceptance (21). The design followed an umbrella review format, chosen to integrate data from systematic, scoping, narrative, and rapid reviews, as well as meta-analyses that focused on AI-based methodologies applied to various vaccine platforms, diseases, and healthcare settings The methodology adhered to the Preferred Reporting Items for Systematic Reviews and Meta-Analyses (PRISMA) 2020 guidelines to ensure methodological rigor, clarity, and replicability (22).

### 2.2 SPIDER framework

The SPIDER tool (Sample, Phenomenon of Interest, Design, Evaluation, Research type) provided the structural basis for defining inclusion criteria (23). This approach facilitated a comprehensive scope of relevant reviews, capturing a wide range of AI applications in vaccine contexts across multiple populations and research designs. (See **Table 1**: SPIDER Tool Components).

**Table 1:** SPIDER tool components.

Component	Description
S (Sample)	Vaccine researchers, healthcare professionals, epidemiologists, AI specialists, regulatory entities, and communities affected by infectious diseases or cancer. Included reviews had to focus on at least one of these stakeholder groups.
PI (Phenomenon of Interest)	Integration of AI (machine learning, deep learning, NLP) in vaccine research, development, distribution, and acceptance.
D (Design)	Systematic, scoping, narrative, rapid reviews, literature reviews, and meta-analyses.
E (Evaluation)	Vaccine efficacy, safety, development timelines, ethical/regulatory concerns, and public acceptance issues informed by AI-based interventions.
R (Research types)	Reviews covering quantitative, qualitative, or mixed-methods studies relevant to AI-driven vaccine development.

### 2.3 Inclusion and exclusion criteria

A set of inclusion and exclusion criteria ensured that only reviews directly addressing AI in vaccine research, distribution logistics, and public acceptance were considered. See **Table 2**. Inclusion and Exclusion Criteria).

**Table 2:** Inclusion and exclusion criteria.

Criteria	Inclusion	Exclusion
Publication Design	Systematic, scoping, narrative, rapid reviews, literature reviews, meta-analyses	Primary research articles, opinion pieces, editorials, conference and abstracts.
Focus	Reviews explicitly covering AI in vaccine R&D, distribution, or acceptance	Reviews lacking AI or focusing exclusively on drug discovery without vaccine relevance
Population	Human vaccines (infectious diseases, cancer) across diverse demographics	Veterinary vaccine reviews, or those with no human vaccine context
Language	Publications in English	Non-English publications

### 2.4 Information sources and search strategy

**Figure 1:**
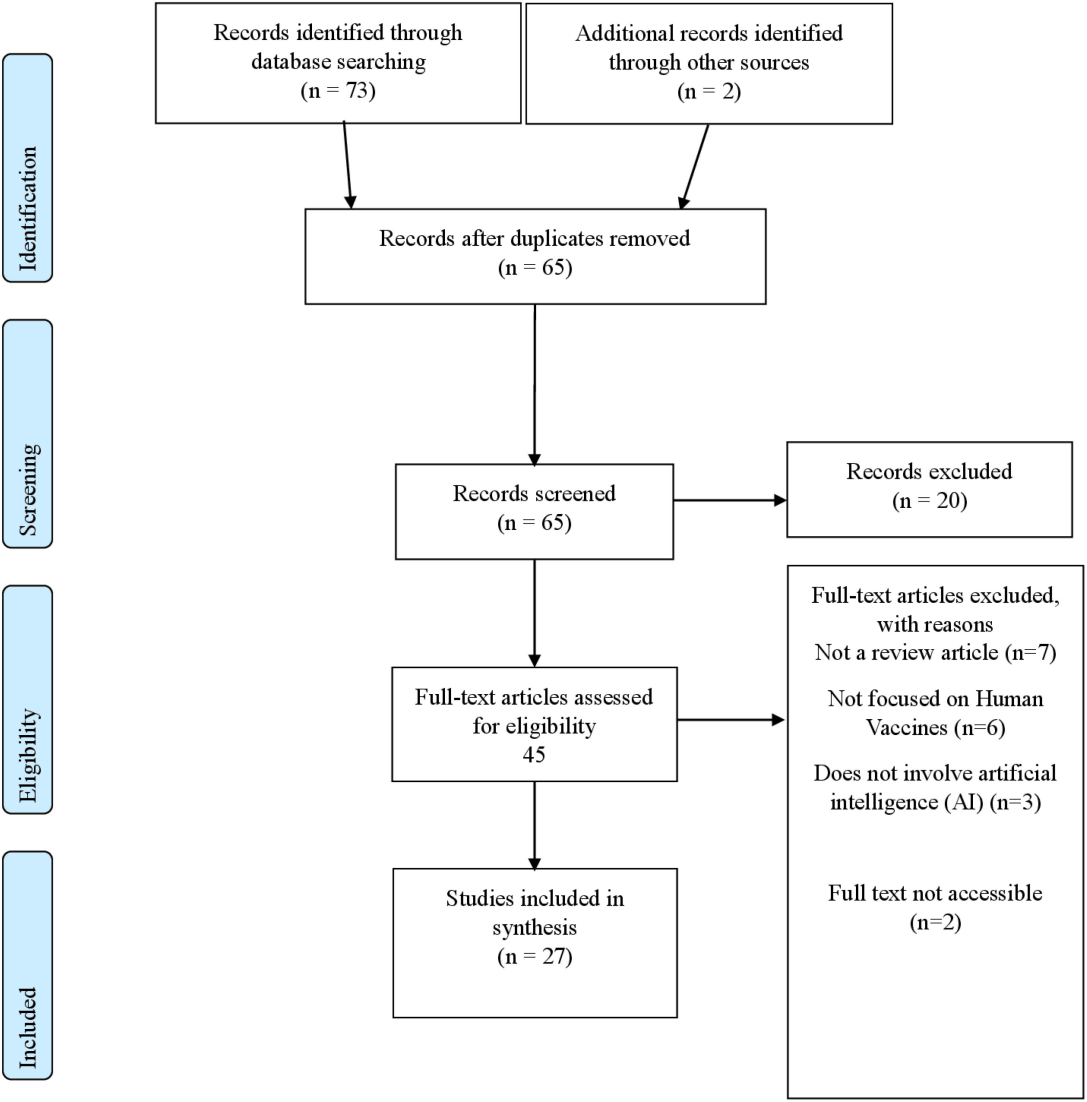
PRISMA flow diagram.

Searches were carried out in PubMed/MEDLINE, Scopus, Web of Science, Embase, and IEEE Xplore to capture all relevant reviews within the established parameters. The time frame was set to capture all relevant reviews published from inception up to January 2025. The Search terms included keywords for AI (machine learning, deep learning, etc.) AND vaccine-related terms (vaccine development, epitope prediction, immunogenicity). (See **Table 3**: Search Terms). The initial number of retrieved references and subsequent exclusions are now detailed in the PRISMA flow diagram (**Figure 1**).

**Table 3:** Search terms.

Search Component	Example Search Terms
AI Concepts	“Artificial Intelligence” OR “AI” OR “Machine Learning” OR “Deep Learning” OR “Natural Language Processing”
Vaccine-Related Terms	“Vaccine Development” OR “Epitope Prediction” OR “Antigen Discovery” OR “Adjuvant Optimization” OR “Clinical Trials”
Disease/Pathogen Focus	“COVID-19” OR “Influenza” OR “HIV” OR “Dengue” OR “Cancer Vaccines” OR “Malaria”
Public Acceptance/Ethical	“Vaccine Hesitancy” OR “Public Sentiment” OR “Ethical Considerations” OR “Regulatory Framework” OR “Data Privacy”
Review Design	“Systematic Review” OR “Scoping Review” OR “Narrative Review” OR “Rapid Review” OR “Literature Review” OR “Meta-Analysis”

### 2.5 Selection process

The records identified through database searches were imported into Rayyan, a systematic review screening tool. Two independent reviewers conducted title and abstract evaluations. Any discrepancies were resolved through discussion or by a third reviewer if necessary.

### 2.6 Data extraction

Data were extracted using a standardized form that was developed and pilot-tested by the review team. Each eligible review was examined for its publication characteristics, methodological approach, AI applications, vaccine platforms, disease targets, and reported outcomes.

### 2.7 Data synthesis

Given the methodological heterogeneity of the included reviews—which encompassed systematic, narrative, scoping, and rapid reviews—a thematic analysis approach was employed to synthesize both qualitative and quantitative data (24).

### 2.8 Risk of bias and quality of assessment

Risk of bias was assessed using the Risk of Bias in Systematic Reviews (ROBIS) tool (25), chosen for its capacity to evaluate different review types, including narrative and scoping reviews. Although primarily intended for systematic reviews, ROBIS offers distinct advantages for appraising diverse methodologies by examining bias across study eligibility criteria, identification and selection processes, data collection and appraisal, and synthesis of findings. Its structured yet adaptable framework allowed us to emphasize transparency and thematic consistency rather than strict meta-analytic components, an important consideration given the methodological variability in the included evidence. We evaluated the methodological quality of each included review using the AMSTAR 2 tool (26), originally designed for systematic reviews of healthcare interventions but here adapted to encompass non-systematic approaches such as narrative, scoping, and rapid reviews.

## 3 Results

This umbrella review encompassed 27 reviews published between 2020 and 2024. The included reviews employed a variety of review methodologies, comprising systematic reviews, narrative reviews, mini reviews, rapid reviews, scoping reviews, and an analytical review. Predominant AI techniques utilized across these studies included machine learning (ML), deep learning (DL), neural networks, natural language processing (NLP), generative models, and network approaches. AI applications identified in the reviewed literature spanned drug design and repurposing, vaccine candidate identification and optimization, epitope prediction, molecular docking, diagnostic and prognostic evaluations, vaccine communication, and public health strategies. The vaccines addressed encompassed mRNA vaccines, peptide-based vaccines, vector-based vaccines, and protein subunit vaccines, targeting diseases such as COVID-19, dengue, influenza, HIV, multidrug-resistant bacteria, and various cancers including Triple-Negative Breast Cancer (TNBC). Detailed characteristics of each included study are provided in **Supplementary Appendix 1**.

### 3.1 Risk of bias and quality assessment

Among the 27 included reviews examined via our adapted ROBIS framework (See **Supplementary Appendix 2**: Risk of Bias), one was judged to have a high overall risk of bias, while the remaining 26 were deemed moderate risk. No reviews received a low overall risk rating. Floresta et al. (2022) (27) exhibited high concern across three critical domains—Eligibility Criteria, Study Selection, and Data Collection & Appraisal—due to the lack of systematic inclusion and exclusion criteria, omission of gray or non-English literature, and absence of a formal bias assessment. Although it offered valuable thematic insights into AI-driven drug design, these multiple methodological omissions significantly compromised confidence in its findings.

Based on our adapted application of AMSTAR 2, the 27 included reviews were distributed across four confidence categories: High (n = 6), Moderate (n = 7), Low (n = 7), and Critically Low (n = 7). (See **Supplementary Appendix 3**: Quality Assessment of included reviews)Overall, the most frequent limitations across the Low and Critically Low reviews were the absence of a registered protocol, non‐systematic search methods, and lack of any formal risk‐of‐bias evaluation. By contrast, the High confidence reviews better adhered to established review standards and more consistently addressed external validation, algorithmic bias, and comprehensive search strategies. The Moderate confidence group occupied an intermediate space: while methodologically sounder than the Low/Critically Low reviews, each had at least one notable gap—often pertaining to reporting transparency or limited exploration of ethical/regulatory concerns. In reconciling the mismatch that arose between ROBIS and AMSTAR 2, we clarify that ROBIS primarily measures risk of bias for systematic methods, while AMSTAR 2 also accounts for reporting standards and overall quality. This explains why a review might be “moderate” under ROBIS but “critically low” under AMSTAR 2 if it lacks protocol registration or formal risk-of-bias assessment.

### 3.2 Thematic synthesis

Our thematic analysis revealed four primary themes: (1) AI Applications in Vaccine Development, (2) Evaluating Impact on Efficacy, Safety, Timelines, (3) Ethical, Logistical, and Regulatory Challenges, and (4) AI-Driven Public Acceptance and Communication. **Table 4** presents a summary of the key findings aligned with the themes.

**Table 4:** Summary of key findings on AI in vaccine development.

Aspect	AI Techniques/Applications	Key Findings	Challenges/Considerations
Antigen & Epitope Prediction	Machine learning, deep learning, reverse vaccinology, epitope mapping	Rapid identification of vaccine targets; optimized candidate selection	Data heterogeneity; model interpretability
Vaccine Design	Multiepitope vaccine design, peptide vaccine design, mRNA optimization	Accelerated design timelines; enhanced immunogenicity	Integration of multi-omics data; formulation challenges
Delivery & Optimization	Nanoparticle design, supply chain modeling	Improved delivery efficiency and vaccine stability	Regulatory hurdles; scalability issues
Clinical & Public Engagement	AI-driven clinical trial optimization, sentiment analysis	Enhanced patient selection; real-time monitoring of public sentiment	Ethical concerns; data privacy

#### 3.2.1 Mapping the landscape of AI applications in vaccine development

##### 3.2.1.1 Diversity of AI methods

All included reviews emphasize ML as the fundamental AI approaches transforming vaccine development. Classical ML techniques—such as random forest (28–32), SVMs (28, 30, 31, 33, 34), gradient boosting (XGBoost) (32, 35), and logistic regression (31)—were employed for tasks including candidate ranking, epitope scoring, and logistic optimization.

Random forest algorithms were among several ML methods employed to expedite vaccine candidate prioritization through comprehensive analyses of immunogenicity and safety profiles (28–31). While not uniquely emphasized, they contributed alongside other methods in evaluating COVID-19 vaccine candidates. Specifically, studies by Floresta et al. (2022) (27), Wang et al. (2021) (29), Lv et al. (2021), and Kaushik et al. (2023) (31) underscored the combined capability of ML techniques—including random forests and neural networks—to rapidly identify promising vaccine candidates with enhanced safety and immune response profiles.

Additionally, multiple ML methods, including SVMs, random forests, and neural networks, significantly contributed to epitope scoring. These approaches advanced the identification of epitopic regions capable of eliciting robust immune responses (30, 32).

Gradient boosting algorithms, especially XGBoost, and logistic regression models proved instrumental in refining vaccine logistics and candidate selection processes. These methods effectively utilized clinical and biological datasets to accurately predict outcomes, enhancing efficiency and decision-making accuracy (36, 37).

Deep Learning (DL) architectures also played a substantial role, particularly convolutional neural networks (CNNs) and recurrent neural networks (RNNs), due to their proficiency in pattern recognition and large-scale data analysis, essential for extracting insights from extensive vaccine-related datasets (27, 31–33, 35, 38–43). Additionally, generative adversarial networks (GANs) and variational autoencoders emerged prominently for novel immunogen design, offering innovative methods to simulate and create potential vaccine candidates (32, 33, 37, 41). Recent developments highlight transformer-based models, known for their superior ability to process sequential biological data, significantly enhancing predictive accuracy in vaccine antigen design.

##### 3.2.1.2 Vaccine platforms and pathogens

mRNA vaccines were frequently discussed, reflecting their prominent role in the COVID-19 pandemic response (28, 42, 43). Beyond mRNA, AI technologies were effectively applied across various vaccine platforms, including viral vectors, protein subunits, peptide-based, and DNA-based vaccines. The scope of pathogens addressed through AI spans influenza, dengue, HIV, multidrug-resistant bacteria, and cancer-specific vaccines. These diverse applications reflect AI’s versatility in handling complexities and unique challenges associated with different pathogens, indicating its broad utility (28, 30, 33, 37, 43–48). Furthermore, AI’s adaptability has expanded to emerging infectious diseases, demonstrating rapid efficacy in response to recent outbreaks. Studies explicitly highlighted AI-driven vaccine designs for pathogens such as the Zika virus and Ebola, emphasizing its critical role in pandemic preparedness (35, 44).

##### 3.2.1.3 Multi-omic integration

A prominent subtheme was the use of multi-omic datasets—including genomic, transcriptomic, proteomic, and immunopeptidomic data—to facilitate the rapid identification of potential antigens (30, 32, 33, 40, 44, 46, 47). For example, multiple reviews described the application of AI in analyzing large viral or tumor datasets to pinpoint structurally conserved epitopes with minimal off-target reactivity (28, 33, 46). Such integrative strategies were reported to accelerate target identification in pathogens with complex genomic architectures such as SARS-CoV-2 and in tumor subtypes such as TNBC, improving the likelihood of finding immunogenic epitopes (30, 38, 43, 47). Additionally, such multi-omic strategies contributed significantly to personalized vaccine design, tailoring immune responses to individual genetic and molecular profiles, further optimizing vaccine efficacy and safety (33). While single-cell multi-omics represents a promising direction, current reviews primarily emphasized general multi-omic integration strategies without explicitly highlighting single-cell approaches prominently.

#### 3.2.2 Evaluating the impact of AI on vaccine efficacy, safety, and timelines

##### 3.2.2.1 Accelerated development timelines

One of the most striking outcomes highlighted across nearly all reviews is how AI drastically reduced vaccine development timelines—a phenomenon most visibly demonstrated by the quick turnaround of COVID-19 vaccines (27, 29, 49). By aiding adaptive clinical trial designs, which incorporate real-time analytics to guide dose adjustments and participant stratification, AI reportedly cut the conventional years R&D cycle down to months in certain pandemic scenarios (37, 39, 44). Moreover, advanced supply chain simulations and downstream process optimizations—such as high-throughput purification—further shortened time-to-market in real-world deployments (50), illustrating that AI’s impact extends beyond epitope selection into every operational facet of vaccine manufacturing. Enhanced AI algorithms have recently enabled even finer predictive modeling for clinical trial outcomes, significantly improving the strategic planning and efficiency of vaccine development pipelines (37, 39).

##### 3.2.2.2 Enhanced efficacy via epitope prediction

Many reviews underscored the role of epitope prediction in improving vaccine efficacy. Tools such as NetMHCpan, DeepVacPred, MARIA, and Vaxign-ML were cited for their ability to correctly rank immunodominant epitopes with high accuracy (27, 30, 32, 39–41). Studies employing these algorithms reported improved immunogenicity in early proof-of-concept experiments, especially in mRNA vaccines targeting the SARS-CoV-2 spike protein (27, 28, 42). Beyond COVID-19, epitope-focused ML pipelines demonstrated promise in designing peptide-based vaccines for HIV and malaria (44, 46), as well as for identifying tumor-specific neoantigens in TNBC (47). Current advancements have integrated AI-driven molecular simulations to further validate and optimize predicted epitopes, ensuring higher clinical translatability.

#### 3.2.3 Ethical, logistical, and regulatory challenges

Across the literature, ethical questions around data privacy, algorithmic bias, and transparency were among the cited concerns (32, 33, 51). Reviews highlighted scenarios where AI might inadvertently exacerbate health inequities if training datasets underrepresent certain ethnic or geographic populations (32, 33). Cold-chain logistics emerged as a central issue for temperature-sensitive platforms such as mRNA vaccines (32, 51).

Regulatory pathways for AI-driven vaccine development remain largely uncharted (37, 44), calling for standardized frameworks and global data-sharing initiatives to ensure AI models are validated effectively and equitably. Recent dialogue emphasizes enhancing regulatory agility and international collaboration to overcome ethical and logistical barriers, ensuring equitable global deployment of AI-powered vaccine solutions (34, 48).

#### 3.2.4 AI-Driven vaccine acceptance and communication

A subset of reviews analyzed vaccine hesitancy and public sentiment NLP approaches (34, 49, 51). By monitoring social media platforms, AI algorithms captured real-time shifts in public sentiment, identified regional “hot spots” of misinformation, and flagged the types of concerns most correlated with reluctance (34, 51). For instance, review linked anxieties over novel mRNA vaccine technology to spikes in social media negativity (34). Chatbots and conversational agents emerged as tools to mitigate hesitancy by providing on-demand, personalized vaccine information (51). Beyond direct communication, AI-based forecasting models were used to guide public health strategies, such as predicting outbreak hotspots, resource allocation, and targeted messaging campaigns (52). These communication-focused AI solutions reflect a broader push to integrate vaccine R&D with public engagement—addressing not only the scientific aspects but also societal factors that influence acceptance and coverage rates.

## 4 Discussion

The integration of AI into vaccine development has emerged as a transformative force, reshaping the traditional paradigms of immunization research and deployment. This umbrella review consolidates evidence from multiple reviews, illustrating AI’s expansive role across the vaccine lifecycle—from initial antigen discovery to public acceptance and pandemic preparedness. The findings reveal not only the profound capabilities of AI in accelerating vaccine innovation but also highlight critical challenges that must be addressed to fully harness its potential.

Across the reviewed literature, AI emerged as a transformative tool in vaccine research and development. For example, one systematic review demonstrated that machine learning techniques enable rapid identification of key vaccine targets – most notably within the SARS-CoV-2 spike protein – thereby streamlining candidate selection (29). Similarly, another study illustrated how deep learning tools facilitate the design of multiepitope vaccines by integrating complex datasets (35). Additional evidence emphasized the efficiency of AI-driven peptide vaccine design through precise epitope prediction (30). while a complementary review showcased the application of AI in reverse vaccinology to prioritize vaccine candidates (32). Collectively, these findings underscore AI’s promise in expediting vaccine development and enhancing immunogenicity, while also highlighting challenges such as data heterogeneity and model interpretability that warrant further investigation.

### 4.1 Comprehensive AI integration across vaccine development

A predominant theme identified is AI’s pervasive application across various stages of vaccine development. ML, DL, NLP, and network-based models have been instrumental in transforming traditional vaccine R&D processes. Classical ML techniques such as random forests, support vector machines (SVMs), gradient boosting, and logistic regression are foundational for tasks like candidate ranking, epitope scoring, and logistic optimization. Concurrently, DL architectures, including CNNs and RNNs, excel in pattern recognition and large-scale data mining, facilitating high-throughput epitope discovery and novel immunogen design through GANs and variational autoencoders. However, the rapid COVID-19 vaccine timeline was multifactorial, with AI acting as one component among several such as government funding, established mRNA technology, and concurrent clinical trials.

Similar integrations have been observed in broader biomedical research, where AI techniques are employed for drug discovery and genomics. For instance, CNNs and RNNs are extensively used in protein structure prediction and genomics, paralleling their application in vaccine epitope prediction (53–55). However, vaccine-specific applications of AI, particularly in multi-omic integration for immunogenicity, remain more specialized, reflecting the unique challenges of vaccine development compared to other biomedical fields (56, 57).

### 4.2 Accelerated timelines and enhanced efficacy

AI’s capacity to drastically reduce vaccine development timelines is one of its most celebrated impacts. The rapid development of COVID-19 vaccines, accelerated from years to months, exemplifies this phenomenon. Nevertheless, attributing the entire COVID-19 acceleration to AI overstates its role. Although AI facilitated real‐time data analysis and rapid epitope predictions, it was government investment, established mRNA platforms, and overlapping clinical trial phases that collectively contributed to reducing the traditional development timeline from years to months. Additionally, AI’s role in high-throughput process development (HTPD) and supply chain optimization has streamlined downstream operations, ensuring rapid scaling and distribution of vaccine candidates.

This acceleration mirrors advancements in oncology, where AI has similarly reduced the time required for biomarker discovery and personalized treatment development (58, 59). However, vaccine development benefits uniquely from AI’s ability to integrate diverse data streams—from genomic sequences to clinical trial data—enabling a more holistic and expedited R&D process. Unlike oncology, which often involves highly individualized treatment protocols, vaccine development benefits from AI’s capacity to generalize across populations, enhancing both speed and scalability.

Furthermore, AI-enhanced epitope prediction tools such as NetMHCpan, DeepVacPred, MARIA, and Vaxign-ML have significantly improved the accuracy of T-cell and B-cell epitope predictions. These tools target highly conserved and immunogenic regions, thereby increasing vaccine efficacy and safety by ensuring robust immune responses while minimizing adverse effects.

Comparable improvements in epitope prediction have been noted in studies focused on autoimmune diseases and infectious diseases beyond COVID-19 (Garcia et al., 2022). However, the integration of AI in predicting immunogenicity for cancer vaccines, such as those targeting TNBC, demonstrates AI’s versatile application in both preventive and therapeutic vaccine strategies.

### 4.3 Ethical, logistical, and regulatory challenges

Despite AI’s transformative potential, several ethical, logistical, and regulatory challenges persist. Ethical concerns such as data privacy, data quality issues, algorithmic biases, and integration challenges—affect vaccine development outcomes. For instance, data heterogeneity can lead to inconsistent model performance, potentially compromising the reliability of AI-driven predictions. Algorithmic biases, arising from underrepresented populations in training datasets, may result in inequitable vaccine efficacy across different demographic groups. Additionally, integration challenges, including interoperability with existing healthcare systems and the need for specialized expertise, can hinder the seamless application of AI technologies in real-world vaccine development scenarios. Addressing these limitations is crucial for ensuring that AI contributions are both effective and equitable.

These ethical concerns are echoed in broader AI applications within healthcare, where similar issues of bias and data privacy are prevalent (60). However, in vaccine development, the stakes are particularly high due to the global scale and public health implications. Unlike more individualized healthcare applications, vaccines must be universally effective and accessible, necessitating a higher standard for ethical AI deployment.

Logistically, managing cold-chain requirements for temperature-sensitive vaccines like mRNA formulations remains a significant hurdle (61). AI-driven supply chain tools offer solutions for optimizing distribution routes and reducing wastage, but these require high-quality, real-time data to be effective (62). Regulatory frameworks for AI-driven vaccine development are still nascent, with limited guidelines from regulatory bodies such as the FDA and EMA (63). The lack of standardized protocols for AI validation in vaccine-related approvals presents a barrier to widespread adoption (64).

### 4.4 AI-driven vaccine acceptance and communication

AI’s role extends beyond technical aspects into public health communication and vaccine acceptance. Sentiment analysis and NLP approaches have been employed to monitor and analyze public sentiment, identifying misinformation and addressing vaccine hesitancy. AI-driven chatbots and conversational agents provide real-time, personalized information, mitigating fears and misconceptions about vaccines. These tools facilitate better public understanding and trust, essential for achieving high vaccination coverage rates. NLP-based sentiment analysis detects misinformation surges and hesitancy trends, yet vaccine decisions often reflect complex socio-cultural factors beyond online sentiment. Some reviews suggest chatbots or automated counseling tools, but the long-term impact on actual uptake is not well established (65).

Furthermore, AI-based forecasting models guide public health strategies by predicting outbreak hotspots, optimizing resource allocation, and enabling targeted messaging campaigns (66, 67). However, the long-term impact of these AI interventions on sustained vaccination behavior and public trust remains uncertain, highlighting the need for longitudinal studies to evaluate their effectiveness over time.

the integration of AI in real-time public sentiment analysis and communication strategies for vaccines represents a more dynamic application, requiring continuous adaptation to evolving public perceptions and misinformation trends (68, 69).

The successful application of AI in the rapid development of COVID-19 vaccines underscores its potential for future pandemic preparedness and outbreak response (70). AI’s ability to rapidly identify and validate vaccine targets, streamline clinical trial processes, and optimize manufacturing and distribution can be replicated for emerging pathogens.

### 4.5 Strengths

This umbrella review consolidates findings from diverse review types (systematic, scoping, narrative, rapid) to provide a comprehensive synthesis of AI applications in vaccine research, development, distribution, and acceptance. By leveraging established tools such as ROBIS and AMSTAR 2 for risk-of-bias and quality assessment, it offers a robust, methodologically transparent appraisal of included evidence. Furthermore, the thematic analysis approach facilitates in-depth exploration of emergent subtopics—such as personalized vaccine design and AI-driven public sentiment analysis—across multiple pathogens and vaccine platforms.

### 4.6 Limitations

Restricting the inclusion to English-language publications may limit global generalizability, particularly for regions where non-English studies predominate. The methodological heterogeneity of the included reviews, which vary from fully systematic to narrative, introduces variability in both reporting quality and analytical rigor. Additionally, the lack of meta-analytic syntheses in the reviews impedes quantitative assessment of pooled outcomes. Moreover, several AI solutions discussed in the included reviews are still in pilot or in silico phases, and their real‐world applicability has yet to be validated in large‐scale clinical trials. Finally, the rapid evolution of AI research raises the possibility that relevant work published after the search cut-off may not be captured. These limitations underscore the need for cautious interpretation of the results and highlight the importance of future research employing more standardized and inclusive methodologies.

## 5 Recommendations

### 5.1 Establish robust data governance and multi-omics integration

To accelerate AI-driven vaccine research, it is imperative to establish robust data governance frameworks and integrate multi-omics data. This can be achieved through the formation of global data-sharing consortia, which facilitate public–private partnerships and international collaborations. By pooling genomic, proteomic, and clinical trial data under harmonized standards, stakeholders can ensure interoperability across diverse vaccine platforms. Implementing standardized data protocols for collection, storage, and sharing will further guarantee data integrity and consistency across various research initiatives.

Investing in secure and scalable cloud infrastructures is essential to support large-scale multi-omics analyses. These infrastructures should incorporate encryption and layered access controls to safeguard data privacy effectively. Additionally, promoting the use of federated learning models will allow AI algorithms to train on decentralized data sources. This approach not only addresses data sovereignty concerns but also enhances collaborative research efforts without compromising data privacy, particularly in low- and middle-income countries.

To mitigate algorithmic bias, it is crucial to embed regular bias audits within AI pipelines. These audits should be conducted by multidisciplinary teams to ensure comprehensive evaluations. Furthermore, training datasets must include under-represented ethnicities and geographic regions to broaden the applicability of AI models and reduce potential biases. By ensuring diverse and inclusive data representation, AI-driven vaccine development can achieve more equitable and effective outcomes, fostering greater public trust and acceptance.

### 5.2 Strengthen regulatory and ethical frameworks for AI in vaccine development

Close collaboration among regulatory bodies (e.g., WHO, FDA, EMA) is vital for clear guidelines on AI-driven clinical trials, data validation, and safety oversight. Transparency requirements regarding model architecture, performance metrics, and interpretability should be standardized before approval.

Ethical review boards (analogous to IRBs) can oversee algorithmic explainability and data governance, while public consultation forums address broader concerns about privacy, digital rights, and data misuse. Global collaboration is crucial for harmonizing legal frameworks, reducing administrative delays—especially during health emergencies.

Concretely, implementing robust bias auditing frameworks and ensuring diverse, representative datasets will enhance AI fairness. Interdisciplinary ethics committees provide additional oversight, maintaining public trust through accountability and transparent communication.

### 5.3 Leverage AI to expedite vaccine timelines and enhance efficacy

Real-time data analytics integrated into clinical trials can dramatically shorten development timelines. Adaptive trial designs, guided by AI, allow prompt dose adjustments and participant stratification. Continuous safety monitoring ensures early identification of adverse events, reducing attrition and improving resource use.

Investment in next-generation epitope prediction tools—especially those based on deep learning—can refine candidate selection while minimizing false positives. Rigorous preclinical validation bridges in silico findings to clinical efficacy. Beyond discovery, AI-based manufacturing simulations help anticipate production bottlenecks, streamline cold-chain logistics, and optimize purification steps. Predictive analytics further aid in forecasting vaccine demand, crucial in low-resource settings.

### 5.4 Build public trust and mitigate vaccine hesitancy through AI-enhanced communication

Real-time sentiment analysis, powered by NLP, can identify misinformation trends and flag vaccine hesitancy hotspots. Sharing anonymized data dashboards with public health authorities supports targeted interventions. Chatbots and conversational agents, if regularly audited, can offer accurate and personalized information, reducing misinformation’s impact.

Community co-creation (involving healthcare workers, religious leaders, civil society organizations) ensures that AI tools align with cultural norms and address local concerns. Clear explanations of AI methodologies help demystify the technology, fostering higher vaccination rates and sustained community engagement.

### 5.5 Prioritize global health equity and future pandemic preparedness

Digital infrastructure must be expanded in underserved regions to ensure equitable access to AI-driven solutions. Investments in internet connectivity, data literacy training, and computational capacity empower local researchers to develop region-specific interventions and bridge the technological divide.

Building local capacity via training programs and technology transfer initiatives fosters long-term self-reliance. AI-driven tools should be designed to run efficiently in resource-limited environments, maximizing their impact in low-resource settings.

Equitable access to AI-based innovations is paramount to ensure underserved populations benefit from advancements in vaccine R&D. Data-sharing partnerships should also extend to regions with fewer resources, enabling them to contribute data and benefit from global health initiatives.

Robust dynamic surveillance systems, integrating real-time epidemiological data with AI modeling, can provide early warnings of emerging threats. Cross-border collaborations—supported by pathogen surveillance networks—facilitate rapid-response strategies during outbreaks. Sustained investment from governments, philanthropic bodies, and industry should fund AI and vaccine research, ensuring flexible, long-term resources that can be swiftly redeployed during health emergencies.

## 6 Implications

The strategic integration of AI across the entire vaccine continuum—from epitope prediction and clinical trial optimization to logistics and public engagement—can profoundly transform global health outcomes. Some included reviews focus on limited interventions—such as small chatbot trials or localized sentiment analyses—and do not constitute conclusive evidence of wide‐scale efficacy. Position AI as a promising but still-emerging tool for pandemic preparedness, rather than a singular solution.

To realize the potential, robust data governance and ethically aligned frameworks must be implemented alongside meaningful stakeholder participation at each phase of vaccine development. Such an approach ensures equitable representation, fosters public trust, and balances accelerated innovation with rigorous safeguards for privacy and safety. Collaboration among healthcare practitioners, policymakers, technology developers, and local communities is essential to streamline development timelines, optimize manufacturing and distribution, and harness AI’s predictive capabilities in outbreak surveillance. By upholding transparent methodologies and cross-sector partnerships, AI-driven vaccine initiatives can not only enhance current immunization efforts but also bolster resilience against future pandemics, reinforcing health security on a global scale.

## 7 Conclusion

The systematic integration of AI into vaccine research, development, distribution, and acceptance has demonstrated notable potential to accelerate discovery timelines, enhance efficacy and safety, and amplify public engagement. By merging advanced ML with multi-omics datasets, stakeholders can pinpoint immunogenic targets and streamline clinical trials. Nevertheless, ethical and regulatory complexities persist—data privacy, algorithmic bias, and equitable global access remain critical concerns. Our review highlights the importance of transparent frameworks, multinational collaboration, and community-driven participatory approaches to fully harness AI’s transformative power. Investments in robust data infrastructures, ethical oversight, and workforce capacity are imperative to ensure these innovations benefit diverse populations. With sustained commitment from governmental, industrial, and academic sectors, AI holds the promise to redefine vaccine development, strengthen pandemic preparedness, and revolutionize global health outcomes.

## Data Availability

The original contributions presented in the study are included in the article/**Supplementary Material**. Further inquiries can be directed to the corresponding author.
