# Supplementary material for: Artificial intelligence in vaccine research and development: an umbrella review
Source: Front Immunol. 2025 May 8;16:1567116. doi: 10.3389/fimmu.2025.1567116 (PMC12095282; doi:10.3389/fimmu.2025.1567116)
Supplement: Supplementary file 1 [file Table1.docx]

Appendix1: Characterestics of included reviews

| Authors (Year) | Study Design | AI Type | AI Applications | Vaccine Type | Target Disease | Key Findings | Strengths/Limitations |
| --- | --- | --- | --- | --- | --- | --- | --- |
| Floresta et al. (2022)(Floresta et al., 2022) | Narrative Review | Machine Learning, Deep Learning | Drug design, vaccine design | NA | COVID-19 | AI significantly accelerated COVID-19 vaccine and drug development processes, identifying promising molecules using de novo and ligand-based drug design techniques. Key results include improved binding affinity predictions and enhanced efficiency in identifying novel therapeutic candidates. | Strengths: Comprehensive review of AI applications in COVID-19; demonstrates potential of AI in accelerating pharmaceutical research. Limitations: Lack of experimental validation data and focus on specific methodologies. |
| Wang et al. (2021)(Wang et al., 2021) | Systematic Review | Machine Learning, Deep Learning | Diagnosis, prognosis evaluation, epidemic trend prediction, drug and vaccine discovery | NA | COVID-19 | AI achieved high accuracy in diagnosis (70%-99.92%), prognosis evaluation (AUC up to 0.997), and epidemic trend prediction. One study identified vaccine targets, including the S protein and nsp3, as potential candidates for COVID-19 vaccine development. AI models also facilitated drug repurposing and discovery. | Strengths: Comprehensive assessment of AI applications in COVID-19; diverse AI applications; insights into future directions. Limitations: High risk of bias in included studies, small sample sizes, limited generalizability. |
| Lv et al. (2021) (Lv et al., 2021) | Narrative Review | Machine Learning, Deep Learning | Drug repurposing, vaccine design | NA | COVID-19 | AI/ML techniques accelerated drug repurposing and vaccine design for COVID-19 by utilizing network-based algorithms, expression-based algorithms, and integrated docking simulations. Identified potential drugs and epitopes, improving efficiency in combating the pandemic. | Strengths: Comprehensive review of AI/ML applications in drug and vaccine discovery; diverse methodologies discussed. Limitations: Lack of systematic criteria for selecting AI/ML algorithms; insufficient experimental validation of predicted candidates. |
| Mohanty & Mohanty (2021)(Mohanty & Mohanty, 2021) | Narrative Review | Machine Learning | Peptide vaccine design, epitope identification | Peptide-based vaccines | RNA viruses | AI aids in identifying immunogenic epitopes and designing peptide vaccines that target conserved viral regions, addressing challenges like high mutation rates in RNA viruses. Highlighted tools include NetMHCpan and DeepVacPred, which enhance epitope prediction and vaccine design efficiency. AI also improves clinical trial recruitment through NLP. | Strengths: Focused on AI's role in tackling RNA virus challenges; comprehensive review of bioinformatics tools. Limitations: Data quality and availability constraints impact AI model accuracy; limited experimental validation of AI predictions. |
| Keshavarzi Arshadi et al. (2020)(Keshavarzi Arshadi et al., 2020) | Mini Review\  Narrative | Machine Learning, Deep Learning | Drug repurposing, vaccine candidate identification, molecular docking, epitope prediction | Various vaccine candidates | COVID-19 | AI models identified molecular targets, optimized epitope predictions, and facilitated drug repurposing efforts for COVID-19. AI-based tools like MARIA and NetMHCpan were highlighted for epitope discovery. Deep Docking identified protease inhibitors. | Strengths: Comprehensive overview of AI's impact on COVID-19 therapeutic research; detailed discussion of computational tools. Limitations: Relies heavily on in silico data, lacking significant experimental validation. |
| Kaushik, Kant, and Christodoulides (2023)(Kaushik et al., 2023) | Mini Review\  Narrative | Machine Learning, Neural Networks | Vaccine candidate identification, antigen detection, reverse vaccinology | Various vaccine candidates | Multidrug-resistant bacteria | AI-driven methods like reverse vaccinology and tools such as VaxiJen, PanRV, and ReVac significantly enhance vaccine development efficiency by identifying antigens and epitopes. AI accelerates vaccine discovery by integrating genomic and proteomic data. | Strengths: Comprehensive review of AI's impact on antimicrobial resistance and vaccine design; highlights innovative AI methodologies. Limitations: Relies heavily on theoretical models with limited experimental validation and user accessibility challenges. |
| Hasanzadeh et al. (2022)(Hasanzadeh et al., 2022) | Narrative Review | Machine Learning, Deep Learning, Neural Networks | Design of nanovectors, CRISPR/Cas enhancement, mRNA vaccine carriers | mRNA vaccines | RNA viruses, COVID-19 | AI optimizes nanovectors for gene delivery, enhances CRISPR/Cas systems for genome editing, and improves mRNA vaccine carrier design. It aids in overcoming extracellular and intracellular barriers and predicts nanoparticle properties. | Strengths: Comprehensive insights into AI applications for gene delivery and vaccine development; multidisciplinary perspective. Limitations: Limited real-world validation; focus on theoretical potential over practical applicatio |
| Arora et al. (2021)(Arora et al., 2021) | Narrative Review | Machine Learning, Deep Learning | Surveillance, diagnosis, drug discovery, vaccine development | mRNA, Vector-based | COVID-19 | AI-based tools significantly enhanced vaccine development, epitope prediction, drug repurposing, and large-scale surveillance. Examples include MARIA and Vaxign-ML for epitope identification. AI improved the efficiency and accuracy of vaccine candidate selection and drug discovery pipelines. | Strengths: Comprehensive coverage of AI applications across multiple domains (surveillance, vaccine, drug discovery). Limitations: Heavy reliance on theoretical frameworks; lacks experimental or real-world validation of the proposed model |
| Goh et al. (2020)(Goh et al., 2020) | Narrative Review | Neural Networks (AI) | Protein disorder analysis for vaccine target identification, viral attenuation design | SARS-CoV-2, mRNA vaccines | SARS-CoV-2 | AI-based protein disorder models identified viral nucleocapsid proteins (N) as promising vaccine targets. The strategy involves mutating regions of disorder to attenuate the virus while maintaining immunogenicity for vaccine development. | Strengths: Innovative use of intrinsic protein disorder in vaccine design; theoretical validation using computational tools. Limitations: Lacks experimental data for validating proposed vaccine candidates; theoretical focus without practical trials. |
| Vaishya et al. (2020)(Vaishya et al., 2020) | Rapid Review | Machine Learning, Neural Networks | Early detection, monitoring, contact tracing, case projection, drug and vaccine development | NA | COVID-19 | AI supports early detection of COVID-19, monitoring treatment, predicting cases and mortality, and accelerating drug and vaccine development through advanced data analysis and neural networks. It reduces healthcare workers' workload and aids prevention strategies. | Strengths: Comprehensive review of AI applications in pandemic management. Limitations: Lack of experimental validation for AI applications; reliance on theoretical frameworks without specific case studies. |
| Naseem et al. (2020)(Naseem et al., 2020) | Scoping Review | Machine Learning, Deep Learning | COVID-19 diagnosis, patient monitoring, drug development | NA | COVID-19 | AI enhanced COVID-19 screening, diagnosis, and patient monitoring, utilizing models such as deep learning for protein structure analysis and drug discovery pipelines. AI-based prediction and contact tracing systems improved epidemic management and resource allocation in LMICs. | Strengths: Focus on LMIC-specific challenges and AI opportunities; application of PRISMA-ScR guidelines. Limitations: Reliance on PubMed database only; lack of experimental validation and potential omission of grey literature. |
| Black et al. (2020)(Black et al., 2020) | Narrative Review | Machine Learning | Accelerated vaccine discovery, reverse vaccinology, adaptive trial design | RNA, Vector-based | COVID-19 | AI technologies, including reverse vaccinology and real-world data integration, drastically reduced vaccine development timelines for COVID-19. Adaptive trials and platform technologies facilitated faster development and approval. | Strengths: Comprehensive discussion on integrating AI in vaccine R&D; practical examples of COVID-19 application. Limitations: Focuses on theoretical advantages; limited real-world validation. |
| Kaushal et al. (2020)(Kaushal et al., 2020) | Systematic Review | Machine Learning, Deep Learning | Drug repurposing, novel drug discovery, vaccine development, antibody generation | NA | COVID-19 | AI enabled faster identification of drugs and vaccine candidates, targeting key SARS-CoV-2 proteins like 3CLpro, ACE2, and TMPRSS2. Approximately 50% of studies focused on 3CLpro, and 16% of identified drugs are in clinical evaluation phases. | Strengths: Comprehensive review of AI applications in COVID-19 therapeutics; highlights practical outcomes. Limitations: Limited focus on experimental validation; theoretical reliance on computational predictions. |
| Cai et al. (2021)(Cai et al., 2021) | Narrative Review | Machine Learning | Epitope prediction for vaccine design, computational methods | mRNA vaccines | COVID-19, Zika, RSV, Influenza | Machine learning enhances epitope prediction accuracy for mRNA vaccines targeting COVID-19 and other infectious diseases. Tools like NetMHCpan and BepiPred improve epitope identification for tailored vaccine development and immunogenicity optimization. | Strengths: Comprehensive analysis of mRNA vaccine mechanisms; highlights computational tools for epitope prediction. Limitations: Emphasis on theoretical predictions without robust experimental validation; limited focus on conformational epitopes. |
| Natali et al. (2021)(Natali et al., 2021) | Narrative Review | Machine Learning, AI for Immune Repertoires | Immune response analysis, vaccine design, antibody discovery | NA | Dengue | AI deconvolutes immune responses, identifies novel antibody candidates, and informs vaccine designs targeting conserved viral regions. Tools like immuneML leverage immune repertoire sequencing to predict antibody specificity and optimize vaccine responses. | Strengths: Integrates high-throughput sequencing data with AI for immune analysis; highlights future research potential. Limitations: Limited focus on practical application; AI models require further experimental validation for clinical impact. |
| Alamoodi et al. (2021)(Alamoodi et al., 2021) | Systematic Review | Sentiment Analysis, Natural Language Processing (NLP) | Analyzing vaccine hesitancy, sentiment polarity, misinformation detection | COVID-19, Measles, HPV | COVID-19, Measles, HPV | Identified public sentiment trends toward vaccination across social media using sentiment analysis. Discussed implications for public health messaging and strategies to counter misinformation. Highlighted sentiment differences by geography and vaccine type. | Strengths: Comprehensive multi-disciplinary approach; systematic literature mapping. Limitations: Focused on English-language studies; relies heavily on computational techniques with limited insights into offline behaviors. |
| Bagabir et al. (2022)(Abubaker Bagabir et al., 2022) | Non Systematic Review | Machine Learning, Deep Learning, AI for Genomic Analysis | Genome sequencing, drug discovery, vaccine development | mRNA, Vector-based | COVID-19 | AI significantly improved the speed and accuracy of identifying SARS-CoV-2 genomic sequences, drug repurposing, and vaccine development. Highlighted the ability of AI to predict mutations and optimize vaccine design against variants like Delta and Omicron. | Strengths: Comprehensive review of AI applications in COVID-19; discusses advantages and challenges. Limitations: Non-systematic methodology; lacks detailed experimental validation and relies on theoretical frameworks. |
| Keulen et al. (2022)(Keulen et al., 2022) | Narrative Review | Machine Learning | Downstream process development, chromatography optimization | Subunit vaccines | General | AI enhances high-throughput process development (HTPD) by optimizing vaccine purification processes, reducing experimental efforts, and improving cost-effectiveness. AI facilitates chromatographic modeling for better antigen purification. | Strengths: Comprehensive review of AI applications in vaccine purification processes; emphasizes practical industrial applications. Limitations: Theoretical focus with limited experimental validation; primarily highlights protein subunit vaccines. |
| Sharma et al. (2022)(Sharma et al., 2022) | Narrative Review | Machine Learning, Deep Learning, AI for Genomic Analysis | Genome sequencing, epitope prediction, mRNA vaccine optimization, clinical trial management | mRNA, Vector-based | COVID-19 | AI tools such as MARIA and NetMHCpan expedited epitope prediction and vaccine candidate selection. AI optimized mRNA sequence design, improved clinical trial efficiency, and reduced vaccine development time to under two years. | Strengths: Comprehensive integration of AI in all phases of vaccine development; detailed case studies on Moderna and Pfizer vaccines. Limitations: Focused primarily on COVID-19; reliance on theoretical frameworks with limited experimental validation. |
| Passanante et al. (2023)(Passanante et al., 2023) | Systematic Review | Conversational AI, NLP | Vaccine communication, combating misinformation, appointment scheduling | COVID-19, HPV | COVID-19, HPV, childhood immunizations | Chatbots have been effective in vaccine communication, reducing vaccine hesitancy by providing timely, credible, and personalized information. However, most studies are exploratory with small sample sizes, limiting generalizability. | Strengths: Comprehensive review of vaccine chatbots; identifies gaps in the literature. Limitations: Small sample sizes; lack of studies on long-term or societal impacts of conversational AI in vaccine communication. |
| Xiao et al. (2023)(Xiao et al., 2023) | Narrative Review | Machine Learning, Network Approaches | Multi-omic immunoprofiling, vaccine response prediction, immune endotyping | mRNA, Protein Subunit | SARS-CoV-2, Influenza, HIV | AI-driven integration of multi-omic datasets enhances vaccine design, enabling precise immune response prediction and identification of novel immune endotypes. Advances in transcriptomics and proteomics offer improved insights into cellular and molecular mechanisms of immunity. | Strengths: Comprehensive review of cutting-edge AI techniques in immunology; focus on multi-omic integration for translational insights. Limitations: Relies on computational frameworks; requires experimental validation to translate findings to clinical applications. |
| Dhanushkumar et al. (2024)(Dhanushkumar et al., 2024) | Narrative Review | Machine Learning, Immunoinformatics | Tumor antigen identification, epitope prediction, personalized vaccine design | Cancer vaccines | Triple-Negative Breast Cancer (TNBC) | AI and multi-omics tools facilitated precise antigen discovery, HLA-binding predictions, and epitope selection for TNBC vaccine development. Highlighted strategies include whole-genome sequencing, RNA sequencing, and predictive algorithms for immune responses. | Strengths: Comprehensive integration of multi-omics and AI approaches; focus on TNBC-specific challenges. Limitations: Heavy reliance on computational predictions; lacks experimental validation. |
| Olawade et al. (2024)(Olawade et al., 2024) | Narrative Review | Machine Learning, Deep Learning, Generative Models, Neural Networks | Antigen selection, epitope prediction, adjuvant discovery, immunogen design | NA | Infectious Diseases | AI revolutionizes vaccine development by streamlining antigen and epitope selection, designing novel immunogens, and optimizing vaccine formulations. Integration of AI and computational tools shortens timelines while improving vaccine safety and efficacy. | Strengths: Comprehensive review of AI applications in vaccine R&D; highlights tools like GANs and MD simulations. Limitations: Lack of detailed real-world case studies; heavy reliance on computational predictions without extensive experimental validation. |
| Zhang et al. (2024)(Zhang et al., 2024) | Narrative Review | Machine Learning, AI for Drug Discovery | Adjuvant discovery, immune modulation, optimization of vaccine formulations | Cancer Vaccines | Various cancers | AI enables the identification of novel adjuvants, including TLR and STING agonists, through high-throughput screening and modeling. It enhances vaccine efficacy by improving immune responses and reducing development costs. | Strengths: Comprehensive overview of AI-driven adjuvant discovery; detailed focus on molecular and cellular mechanisms. Limitations: Lack of experimental validation; primarily theoretical focus with minimal practical application data. |
| Rouhani & Mozaffari (2024)(Rouhani & Mozaffari, 2024) | Analytical review | Topic Modeling, Sentiment Analysis, Classification | Research topic modeling, sentiment analysis, topic classification | NA | COVID-19 | Using topic modeling and sentiment analysis, eight main research areas related to COVID-19 vaccines were identified, including reporting, acceptance, and vaccine reactions. A CNN-LSTM model predicted topic classification with 75% accuracy. | Strengths: Novel combination of AI techniques for systematic literature review; provides actionable insights for future research. Limitations: Limited to articles from Scopus and PubMed; relies on lexicon-based sentiment analysis. |
| Asediya et al. (2024)(Asediya et al., 2024) | Narrative Review | Machine Learning, Deep Learning, Natural Language Processing | Protein structure prediction, epitope prediction, adjuvant discovery, vaccine optimization, supply chain modeling | mRNA, Viral Vector, Protein Subunit, DNA | Various Infectious Diseases | AI accelerates vaccine development through protein structure prediction tools (AlphaFold), adjuvant discovery, and supply chain optimization. AI also enhances vaccine formulations and expedites clinical trials through advanced modeling and simulation. | Strengths: Broad coverage of AI applications in vaccine development; includes real-world examples like AlphaFold and vaccine design tools. Limitations: Lacks detailed experimental validation; emphasizes theoretical capabilities without extensive practical case studies. |
| Kumar et al. (2024)(Kumar et al., 2024) | Scoping Review | Machine Learning, Deep Learning | Epitope design, neoantigen prediction, MHC-peptide binding prediction, personalized vaccine design | mRNA, DNA, Peptide-based | Various Cancers | AI enhances the precision of cancer vaccine design by optimizing epitope prediction and MHC-peptide binding, enabling personalized immunotherapies. Tools like DiscoTope-3.0 address limitations in epitope mapping and antigen analysis. | Strengths: Comprehensive discussion of AI's impact on vaccine personalization; integration of bioinformatics and immunomics tools. Limitations: Relies on computational predictions without robust experimental validation; ethical and regulatory concerns remain. |

References

Abubaker Bagabir, S., Ibrahim, N. K., Abubaker Bagabir, H., & Hashem Ateeq, R. (2022). Covid-19 and Artificial Intelligence: Genome sequencing, drug development and vaccine discovery. *Journal of Infection and Public Health*, *15*(2), 289–296. https://doi.org/10.1016/J.JIPH.2022.01.011

Alamoodi, A. H., Zaidan, B. B., Al-Masawa, M., Taresh, S. M., Noman, S., Ahmaro, I. Y. Y., Garfan, S., Chen, J., Ahmed, M. A., Zaidan, A. A., Albahri, O. S., Aickelin, U., Thamir, N. N., Fadhil, J. A., & Salahaldin, A. (2021). Multi-perspectives systematic review on the applications of sentiment analysis for vaccine hesitancy. *Computers in Biology and Medicine*, *139*, 104957. https://doi.org/10.1016/J.COMPBIOMED.2021.104957

Arora, G., Joshi, J., Mandal, R. S., Shrivastava, N., Virmani, R., & Sethi, T. (2021). Artificial intelligence in surveillance, diagnosis, drug discovery and vaccine development against covid-19. *Pathogens*, *10*(8), 1048. https://doi.org/10.3390/PATHOGENS10081048/S1

Asediya, V. S., Anjaria, P. A., Mathakiya, R. A., Koringa, P. G., Nayak, J. B., Bisht, D., Fulmali, D., Patel, V. A., & Desai, D. N. (2024). Vaccine development using artificial intelligence and machine learning: A review. *International Journal of Biological Macromolecules*, *282*, 136643. https://doi.org/10.1016/J.IJBIOMAC.2024.136643

Black, S., Bloom, D. E., Kaslow, D. C., Pecetta, S., & Rappuoli, R. (2020). Transforming vaccine development. *Seminars in Immunology*, *50*, 101413. https://doi.org/10.1016/J.SMIM.2020.101413

Cai, X., Li, J. J., Liu, T., Brian, O., & Li, J. (2021). Infectious disease mRNA vaccines and a review on epitope prediction for vaccine design. *Briefings in Functional Genomics*, *20*(5), 289–303. https://doi.org/10.1093/BFGP/ELAB027

Dhanushkumar, T., M E, S., Selvam, P. K., Rambabu, M., Dasegowda, K. R., Vasudevan, K., & George Priya Doss., C. (2024). Advancements and hurdles in the development of a vaccine for triple-negative breast cancer: A comprehensive review of multi-omics and immunomics strategies. *Life Sciences*, *337*, 122360. https://doi.org/10.1016/J.LFS.2023.122360

Floresta, G., Zagni, C., Gentile, D., Patamia, V., & Rescifina, A. (2022). Artificial Intelligence Technologies for COVID-19 De Novo Drug Design. *International Journal of Molecular Sciences*, *23*(6). https://doi.org/10.3390/IJMS23063261

Goh, G. K. M., Dunker, A. K., Foster, J. A., & Uversky, V. N. (2020). A Novel Strategy for the Development of Vaccines for SARS-CoV-2 (COVID-19) and Other Viruses Using AI and Viral Shell Disorder. *Journal of Proteome Research*, *19*(11), 4355–4363. https://doi.org/10.1021/ACS.JPROTEOME.0C00672

Hasanzadeh, A., Hamblin, M. R., Kiani, J., Noori, H., Hardie, J. M., Karimi, M., & Shafiee, H. (2022). Could artificial intelligence revolutionize the development of nanovectors for gene therapy and mRNA vaccines? *Nano Today*, *47*, 101665. https://doi.org/10.1016/J.NANTOD.2022.101665

Kaushal, K., Sarma, P., Rana, S. V., Medhi, B., & Naithani, M. (2020). Emerging role of artificial intelligence in therapeutics for COVID-19: a systematic review. *Journal of Biomolecular Structure & Dynamics*, *40*(10), 1. https://doi.org/10.1080/07391102.2020.1855250

Kaushik, R., Kant, R., & Christodoulides, M. (2023). Artificial intelligence in accelerating vaccine development - current and future perspectives. *Frontiers in Bacteriology*, *2*, 1258159. https://doi.org/10.3389/FBRIO.2023.1258159

Keshavarzi Arshadi, A., Webb, J., Salem, M., Cruz, E., Calad-Thomson, S., Ghadirian, N., Collins, J., Diez-Cecilia, E., Kelly, B., Goodarzi, H., & Yuan, J. S. (2020). Artificial Intelligence for COVID-19 Drug Discovery and Vaccine Development. *Frontiers in Artificial Intelligence*, *3*, 560670. https://doi.org/10.3389/FRAI.2020.00065/BIBTEX

Keulen, D., Geldhof, G., Bussy, O. Le, Pabst, M., & Ottens, M. (2022). Recent advances to accelerate purification process development: A review with a focus on vaccines. *Journal of Chromatography A*, *1676*, 463195. https://doi.org/10.1016/J.CHROMA.2022.463195

Kumar, A., Dixit, S., Srinivasan, K., M, D., & Vincent, P. M. D. R. (2024). Personalized cancer vaccine design using AI-powered technologies. *Frontiers in Immunology*, *15*, 1357217. https://doi.org/10.3389/FIMMU.2024.1357217/BIBTEX

Lv, H., Shi, L., Berkenpas, J. W., Dao, F. Y., Zulfiqar, H., Ding, H., Zhang, Y., Yang, L., & Cao, R. (2021). Application of artificial intelligence and machine learning for COVID-19 drug discovery and vaccine design. *Briefings in Bioinformatics*, *22*(6). https://doi.org/10.1093/BIB/BBAB320

Mohanty, E., & Mohanty, A. (2021). Role of artificial intelligence in peptide vaccine design against RNA viruses. *Informatics in Medicine Unlocked*, *26*. https://doi.org/10.1016/J.IMU.2021.100768

Naseem, M., Akhund, R., Arshad, H., & Ibrahim, M. T. (2020). Exploring the Potential of Artificial Intelligence and Machine Learning to Combat COVID-19 and Existing Opportunities for LMIC: A Scoping Review. *Journal of Primary Care & Community Health*, *11*. https://doi.org/10.1177/2150132720963634

Natali, E. N., Babrak, L. M., & Miho, E. (2021). Prospective Artificial Intelligence to Dissect the Dengue Immune Response and Discover Therapeutics. *Frontiers in Immunology*, *12*, 574411. https://doi.org/10.3389/FIMMU.2021.574411/BIBTEX

Olawade, D. B., Teke, J., Fapohunda, O., Weerasinghe, K., Usman, S. O., Ige, A. O., & Clement David-Olawade, A. (2024). Leveraging artificial intelligence in vaccine development: A narrative review. *Journal of Microbiological Methods*, *224*, 106998. https://doi.org/10.1016/J.MIMET.2024.106998

Passanante, A., Pertwee, E., Lin, L., Lee, K. Y., Wu, J. T., & Larson, H. J. (2023). Conversational AI and Vaccine Communication: Systematic Review of the Evidence. *J Med Internet Res 2023;25:E42758 Https://Www.Jmir.Org/2023/1/E42758*, *25*(1), e42758. https://doi.org/10.2196/42758

Rouhani, S., & Mozaffari, F. (2024). Comprehensive analytics of COVID-19 vaccine research: From topic modeling to topic classification. *Artificial Intelligence in Medicine*, *157*, 102980. https://doi.org/10.1016/J.ARTMED.2024.102980

Sharma, A., Virmani, T., Pathak, V., Sharma, A., Pathak, K., Kumar, G., & Pathak, D. (2022). Artificial Intelligence-Based Data-Driven Strategy to Accelerate Research, Development, and Clinical Trials of COVID Vaccine. *BioMed Research International*, *2022*, 7205241. https://doi.org/10.1155/2022/7205241

Vaishya, R., Javaid, M., Khan, I. H., & Haleem, A. (2020). Artificial Intelligence (AI) applications for COVID-19 pandemic. *Diabetes & Metabolic Syndrome: Clinical Research & Reviews*, *14*(4), 337–339. https://doi.org/10.1016/J.DSX.2020.04.012

Wang, L., Zhang, Y., Wang, D., Tong, X., Liu, T., Zhang, S., Huang, J., Zhang, L., Chen, L., Fan, H., & Clarke, M. (2021). Artificial Intelligence for COVID-19: A Systematic Review. *Frontiers in Medicine*, *8*, 704256. https://doi.org/10.3389/FMED.2021.704256/BIBTEX

Xiao, H., Rosen, A., Chhibbar, P., Moise, L., & Das, J. (2023). From bench to bedside via bytes: Multi-omic immunoprofiling and integration using machine learning and network approaches. *Human Vaccines & Immunotherapeutics*, *19*(3), 2282803. https://doi.org/10.1080/21645515.2023.2282803

Zhang, W. Y., Zheng, X. L., Coghi, P. S., Chen, J. H., Dong, B. J., & Fan, X. X. (2024). Revolutionizing adjuvant development: harnessing AI for next-generation cancer vaccines. *Frontiers in Immunology*, *15*, 1438030. https://doi.org/10.3389/FIMMU.2024.1438030/BIBTEX
