# Supplementary material for: Artificial intelligence in vaccine research and development: an umbrella review
Source: Front Immunol. 2025 May 8;16:1567116. doi: 10.3389/fimmu.2025.1567116 (PMC12095282; doi:10.3389/fimmu.2025.1567116)
Supplement: Supplementary file 2 [file Table2.docx]

Appendix 2: Risk of Bias of included reviews

| Author, Year | Phase 1 Relevance | Domain 1: Eligibility Criteria | Domain 2: Study Selection | Domain 3: Data Collection and Appraisal | Domain 4: Synthesis and Findings | Overall Risk of Bias | Comments |
| --- | --- | --- | --- | --- | --- | --- | --- |
| Floresta et al., 2022 | Yes: Relevant to AI in COVID-19 de novo drug design | HC: Inclusion/exclusion criteria not clearly outlined as systematic | HC: No mention of non-English/gray literature; no formal search protocol | HC: No risk-of-bias assessment reported; limited clarity on data extraction | MC: Offers thematic synthesis but lacks robust quantitative methods | High | Multiple major gaps (no systematic protocol, no bias assessment). Provides valuable AI insights but missing critical methodological components. |
| Wang et al., 2021 | Yes: Systematic review on AI for COVID-19 | LC: Clearly defined inclusion/exclusion criteria | LC: Comprehensive database search following PRISMA guidelines | MC: Used PROBAST for diagnostic/prognostic but limited depth for other areas | MC: Primarily descriptive; lacks meta-analysis for quantitative pooling | Moderate | Robust coverage of AI applications with PRISMA compliance, but would benefit from deeper bias evaluation in non-diagnostic studies and potential meta-analytic integration. |
| Lv et al., 2021 | Yes: Systematic review of AI/ML for COVID-19 | LC: Criteria based on PRISMA, well-defined | LC: Searched five databases (PubMed, Google Scholar, etc.) systematically | MC: Standardized extraction but no detailed bias assessment | MC: Descriptive synthesis only; lacks meta-analysis | Moderate | Provides extensive AI/ML insights but needs explicit risk-of-bias appraisal and quantitative integration for stronger validity. |
| Mohanty & Mohanty, 2021 | Yes: Focuses on AI in peptide vaccine design | LC: Criteria focusing on peptide vaccines + AI applications | MC: Search strategy not fully detailed; databases utilized but not all | MC: Standard approach yet no explicit mention of bias assessment | MC: Descriptive with no meta-analysis or quantitative approach | Moderate | Valuable overview of AI in peptide vaccine design. Greater clarity on bias appraisal and more rigorous search details would strengthen the findings. |
| Keshavarzi Arshadi et al., 2020 | Yes: AI in COVID-19 drug/vaccine discovery | LC: Clearly defined AI-based criteria for COVID-19 | MC: Broad search, unclear comprehensiveness (no mention of non-English) | MC: Standardized data but no detailed risk-of-bias assessment | MC: Thematic synthesis; lacks quantitative pooling | Moderate | Highlights AI for drug/vaccine development effectively but needs clearer coverage of database scope and a more explicit bias assessment. |
| Kaushik et al., 2023 | Yes: AI’s role in vaccine dev. for antimicrob. resist. | LC: Well-defined criteria based on AI relevance | MC: Key databases searched, but non-English or gray literature not covered | MC: Limited mention of risk-of-bias methods | MC: Primarily descriptive, no meta-analysis | Moderate | Mini‐review effectively discusses reverse vaccinology; improved bias assessment and broader database coverage would enhance reliability. |
| Hasanzadeh et al., 2022 | Yes: AI for nanovectors in gene therapy/mRNA vax | LC: Focus on AI + nanovectors is clear | MC: Broad search, uncertain about coverage of non-English sources | MC: No explicit bias‐assessment protocol | MC: Descriptive approach only; no quantitative or meta‐analysis | Moderate | Useful detail on AI-driven gene delivery optimization; formal bias appraisal needed to bolster methodological rigor. |
| Arora et al., 2021 | Yes: AI in COVID-19 (surveillance, diagnosis) | LC: Focus on AI solutions is clearly defined | MC: Broad search but minimal mention of non-English or gray lit. | MC: Standardized data collection but no formal bias tool used | MC: Thematic synthesis with no meta-analysis | Moderate | Strong overview of AI across multiple COVID-19 domains; a formal risk-of-bias tool would raise confidence in findings. |
| Goh et al., 2020 | Yes: AI for COVID-19 vaccine dev. via shell disorder | LC: Clear focus on AI + disorder-based models | MC: Search strategy documented but excludes non-English/gray coverage | MC: No explicit bias assessment; moderate detail on data extraction | MC: Descriptive synthesis only; no quantitative analysis | Moderate | Novel AI application for vaccine dev.; stronger bias appraisal, broader search, and possible meta-analytic approach would elevate rigor. |
| Vaishya et al., 2020 | Yes: AI for COVID-19 diagnosis, treatment, vax | MC: Inclusion/exclusion present but not deeply explained | MC: Uses PubMed, Scopus, Google Scholar; no mention of non-English | MC: No formal bias assessment; data collection described but not standardized fully | MC: Thematic summary only; lacks quantitative methods | Moderate | Identifies broad AI applications in the pandemic. A formal bias assessment, plus extended search coverage, would strengthen conclusions. |
| Naseem et al., 2020 | Yes: AI/ML for COVID-19 in LMICs | LC: PRISMA-ScR with well-defined criteria | MC: PubMed only; exclusion of non-English/gray possible | MC: Structured data extraction but limited bias assessment | MC: Descriptive with no meta-analysis | Moderate | Valuable scoping review for LMIC context. Expanding databases, incorporating gray lit, and adding bias assessment would enhance generalizability. |
| Black et al., 2020 | Yes: AI advanced tech. in vaccine dev. | LC: Focus on tech & method is clear | MC: Search strategy not well specified; no mention of non-English | MC: Thematic approach; no explicit risk-of-bias method | MC: Descriptive narrative only; lacks meta-analysis | Moderate | Provides forward-looking insights on AI in vaccine R&D. A more systematic approach + formal bias appraisal would improve reliability. |
| Kaushal et al., 2020 | Yes: AI for COVID-19 therapeutics | LC: Defined inclusion/exclusion (PRISMA-based) | MC: Broad coverage of major databases but excludes non-English, gray lit. | MC: Standard extraction but no explicit bias tool used | MC: Descriptive with no meta-analysis | Moderate | Explores AI-driven approaches in drug/vaccine dev. thoroughly. Detailed bias evaluation and quantitative synthesis would add depth. |
| Cai et al., 2021 | Yes: mRNA vaccines & computational epitope prediction | LC: Clear bioinformatics + vaccine design focus | MC: Search strategy not fully detailed; excludes gray/non-English likely | MC: Computational emphasis; no explicit risk-of-bias approach | MC: Narrative overview; no quantitative analysis | Moderate | Comprehensive coverage of mRNA vaccine tools. Formal bias assessment and a more transparent search strategy would bolster confidence. |
| Natali et al., 2021 | Yes: AI for dengue immunology & therapy discovery | MC: Criteria not explicitly described | MC: Search comprehensiveness unclear; minimal mention of non-English | MC: Standardized data extraction but no bias assessment | MC: Thematic focus on AI potential; lacks quantitative integration | Moderate | Demonstrates AI’s role in dengue vaccine dev., yet requires more explicit eligibility criteria and a formal bias assessment to achieve lower risk-of-bias. |
| Alamoodi et al., 2021 | Yes: Sentiment analysis for vaccine hesitancy | LC: PRISMA-based inclusion/exclusion, well-defined focus | LC: Comprehensive search (6 databases) but no non-English details | MC: Standard extraction; no explicit bias tool used | MC: Thematic across social/medical domains; lacks meta-analysis | Moderate | Offers valuable multi-perspective insights into hesitancy. Explicit bias evaluation and expanding geographic scope might strengthen the evidence base. |
| Bagabir et al., 2022 | Yes: AI for genomic seq., drug/vax dev. (COVID-19) | MC: Criteria not explicitly described | MC: Non-systematic approach; excludes non-English/gray coverage possibly | MC: Summaries lack formal bias method | MC: Descriptive across themes; lacks quantitative integration | Moderate | Highlights key AI tools but is methodologically limited (non-systematic approach, no bias appraisal). More rigorous methods are needed. |
| Keulen et al., 2022 | Yes: Vaccine purification & AI downstream processes | MC: General inclusion criteria, not systematically elaborated | MC: No comprehensive, systematic database detail | MC: Discusses data-driven modeling but no bias assessment | MC: Synthesis includes practical examples; lacks statistical validation | Moderate | Detailed account of AI in downstream processes. A structured search and formal bias appraisal would raise overall reliability. |
| Sharma et al., 2022 | Yes: AI in vaccine R&D & trials (COVID-19) | MC: Criteria discussed but minimal detail on systematic approach | MC: Limited info on database comprehensiveness or gray lit. | MC: No formal bias assessment method | MC: Comprehensive thematic discussion; lacks quantitative validation | Moderate | Showcases AI in R&D, but explicit risk-of-bias evaluation and more robust search coverage are needed for stronger evidence. |
| Passanante et al., 2023 | Yes: Conversational AI for vaccine communication | LC: Inclusion/exclusion via PRISMA, well-defined | LC: Comprehensive search (13 databases, gray lit, preprints) | MC: Standard data extraction; no formal bias assessment of included studies | MC: Narrative synthesis only, no statistical integration | Moderate | Significant insights into vaccine chatbots. Adding a formal risk-of-bias step and quantitative approaches could enhance confidence in the findings. |
| Xiao et al., 2023 | Yes: ML + multi-omic immunoprofiling | LC: Clearly focused on multi-omic data integration for vaccine/therapy | MC: Systematic selection details unclear; no mention of gray or non-English | MC: Discusses ML models but no formal bias assessment | MC: Thematic focus on advanced methodologies; no quantitative pooling | Moderate | Integrates ML + omics effectively. Would benefit from explicit bias appraisal and systematic coverage of all relevant databases. |
| Dhanushkumar et al., 2024 | Yes: TNBC vaccine dev. w/ multi-omics + immunoinformatics | MC: Inclusion/exclusion not explicitly systematic | MC: Method lacks detailed search strategy or mention of gray literature | MC: No explicit bias assessment for included tools/datasets | MC: Comprehensive narrative on epitope prediction but no quantitative approach | Moderate | Effectively explores AI for TNBC vaccines. Clarifying systematic methods and bias evaluation would make the conclusions more robust. |
| Olawade et al., 2024 | Yes: Transformative AI in vaccine design | LC: Inclusion criteria focused on AI-based vaccine dev. approaches | MC: Multiple databases but excludes non-English/gray | MC: Standard data collection; no formal risk-of-bias approach | MC: Thematic synthesis with no quantitative validation | Moderate | Strong narrative on epitope prediction/adjuvant design. Needs systematic method details, explicit bias appraisal, and potential quantitative integration. |
| Zhang et al., 2024 | Yes: AI in adjuvant dev. for cancer vaccines | MC: AI-based screening focus but lacks explicit eligibility criteria | MC: Limited database/selection details; no mention of gray lit. | MC: Comprehensive discussion but no formal bias assessment | MC: Narrative on AI-based adjuvant discovery; no statistical validation | Moderate | Insightful analysis of AI’s role in cancer vaccine adjuvants. A more systematic search and bias appraisal would enhance methodological rigor. |
| Rouhani & Mozaffari, 2024 | Yes: Topic modeling & sentiment analysis (COVID-19) | MC: Inclusion restricted to Scopus/PubMed; no non-English or gray lit. | MC: Focused search strategy but lacks transparency | MC: Standard use of LDA, sentiment analysis; no bias tool | MC: Thematic synthesis with advanced analytics; no quantitative validation | Moderate | Innovatively combines NLP & ML for vaccine research. Broader search coverage, a formal bias assessment, and meta-analytic approaches would improve reliability. |
| Asediya et al., 2024 | Yes: Comprehensive AI in vaccine dev. | MC: Inclusion criteria general, lacking systematic detail | MC: Database strategy not fully transparent | MC: Discusses AI models; no formal bias assessment | MC: Descriptive/thematic with no meta-analysis | Moderate | Excellent overview of AI in vaccine dev. but limited clarity on search methods and no formal bias tool. Statistical or meta-analytic integration recommended. |
| Kumar et al., 2024 | Yes: AI integration in personalized cancer vaccines | LC: Inclusion/exclusion per PRISMA-ScR guidelines | LC: Comprehensive search (PubMed, Scopus, Cochrane) with potential for gray lit. | MC: Standardized data extraction but no formal bias assessment | MC: Thematic synthesis highlighting AI apps; lacks quantitative validation | Moderate | Detailed exploration of personalized vaccine design. Would benefit from a formal risk-of-bias tool and potential meta-analysis for stronger evidence synthesis. |

**Abbreviation Legend**

**LC (Low Concern):** Little to no concern identified in this domain; the review meets most recommended practices.

**MC (Moderate Concern):** Some limitations present (e.g., incomplete search coverage, no formal bias tool), but not severe enough to wholly undermine confidence.

**HC (High Concern):** Significant omissions or flaws (e.g., no clear criteria, no mention of any search strategy or bias assessment), likely compromising the domain’s validity
