# Supplementary material for: Artificial intelligence in vaccine research and development: an umbrella review
Source: Front Immunol. 2025 May 8;16:1567116. doi: 10.3389/fimmu.2025.1567116 (PMC12095282; doi:10.3389/fimmu.2025.1567116)
Supplement: Supplementary file 3 [file Table3.docx]

Appendix3: Quality Assessment of the included reviews

| Authors (year) | Overall Quality | Key Limitations |
| --- | --- | --- |
| Floresta et al. (2022) | Critically Low | Lacks systematic search strategy; no risk of bias assessment; limited methodological transparency. |
| Wang et al. (2021) | High | High heterogeneity in included studies; limited focus on algorithmic bias and external validation. |
| Lv et al. (2021) | Moderate Confidence | Lack of systematic screening for candidate drugs; insufficient discussion of algorithm selection criteria; limited evaluation of experimental reliability |
| Mohanty & Mohanty (2021) | Critically Low | Non-systematic approach; lacks explicit methodology; limited generalizability due to narrow focus. |
| Keshavarzi Arshadi et al. (2020) | Moderate Confidence | Limited exploration of real-world application challenges; lack of systematic bias evaluation; insufficient discussion on dataset quality and representativeness |
| Kaushik et al. (2023) | Moderate Confidence | Limited discussion on the challenges of real-world deployment; reliance on in silico methods without experimental validation; minimal coverage of algorithmic biases and dataset quality |
| Hasanzadeh et al. (2022) | Critically Low | Lacks systematic methodology; no explicit risk of bias assessment; limited discussion on heterogeneity and reproducibility. |
| Arora et al. (2021) | Critically Low | Non-systematic approach; lacks transparent methodology; insufficient exploration of dataset biases and algorithmic limitations. |
| Goh et al. (2020) | Critically Low | Lacks systematic methodology; insufficient discussion of real-world challenges and ethical implications; no explicit quality appraisal. |
| Vaishya et al. (2020) | Low Confidence | Limited methodological rigor; rapid review approach lacks systematic evaluation; minimal discussion on data quality and algorithmic biases |
| Naseem et al. (2020) | Moderate Confidence | Limited database coverage (PubMed only); potential exclusion of gray literature; insufficient discussion of ethical and regulatory challenges |
| Black et al. (2020) | Low Confidence | Non-systematic approach; no explicit quality appraisal or exploration of ethical considerations. |
| Kaushal et al. (2020) | High Confidence | High heterogeneity among included studies; lacks detailed exploration of dataset biases. |
| Cai et al. (2021) | Moderate Confidence | Lacks systematic methodology; minimal discussion of long-term impacts and validation. |
| Natali et al. (2021) | Critically Low | Non-systematic review; lacks explicit quality assessment and methodological transparency. |
| Alamoodi et al. (2021) | High Confidence | High variability in included studies; minimal exploration of long-term impacts of interventions. |
| Bagabir et al. (2022) | Critically Low | Non-systematic approach; lacks explicit methodology and quality appraisal. |
| Keulen et al. (2022) | High Confidence | Lacks systematic evaluation of real-world implementation challenges; limited discussion on data representativeness and ethical considerations |
| Sharma et al. (2022) | Low Confidence | Non-systematic approach; lacks explicit appraisal of included data and validation. |
| **Passanante et al. (2023)**​ | Moderate Confidence | Small sample sizes in studies reviewed; lacks long-term impact analysis; limited geographic diversity. |
| **Xiao et al. (2023)**​ | High Confidence | Limited discussion on ethical implications and real-world implementation; no pre-registered protocol. |
| **Dhanushkumar et al. (2024)**​ | Moderate Confidence | Lack of explicit bias assessment; limited focus on heterogeneity and generalizability. |
| **Olawade et al. (2024)**​ | Low Confidence | Non-systematic review; lacks detailed risk of bias assessment; insufficient methodological transparency. |
| **Zhang et al. (2024)**​ | Low Confidence | No explicit quality assessment; insufficient exploration of real-world challenges and heterogeneity. |
| **Rouhani et al. (2024)**​ | Low Confidence | No pre-registered protocol; lacks systematic quality assessment; limited discussion of external validity. |
| **Asediya et al. (2024)**​ | Low Confidence | Non-systematic methodology; insufficient detail on data quality and generalizability. |
| **Kumar et al. (2024)**​ | High Confidence | Limited focus on ethical implications; lacks explicit quality appraisal for included datasets. |
